# Oral Health Status among Migrants from Middle- and Low-Income Countries to Europe: A Systematic Review

**DOI:** 10.3390/ijerph182212203

**Published:** 2021-11-20

**Authors:** Dorina Lauritano, Giulia Moreo, Francesco Carinci, Vincenzo Campanella, Fedora Della Vella, Massimo Petruzzi

**Affiliations:** 1 Department of Translational Medicine and for Romagna, University of Ferrara, 44121 Ferrara, Italy; crc@unife.it; 2 Dental and Maxillo-Facial Surgery Unit, IRCCS Ca’ Granda Ospedale Maggiore Policlinico di Milano, 20122 Milan, Italy; giulia.moreo@unimi.it; 3 Department of Clinical Science and Translational Medicine, University of Rome “Tor Vergata”, 00113 Rome, Italy; vincenzo.campanella@uniroma2.it; 4 Interdisciplinary Department of Medicine, University of Bari, 70121 Bari, Italy; dellavellaf@gmail.com (F.D.V.); massimo.petruzzi@uniba.it (M.P.)

**Keywords:** oral health, migrants, oral health inequalities, migration to Europe, socioeconomic status

## Abstract

Introduction. Economic inequality, political instability and globalization have contributed to the constant growth of the migration phenomenon in recent years. In particular, a total of 4.2 million people migrated to Europe during 2019 and most of them settled in Germany, France and Italy. Objectives. The objective of this study was to conduct a systematic review of studies analyzing the oral health condition among migrants from middle- and low-income countries to Europe and assessing the eventual association between their sociodemographic and socioeconomic characteristics and oral health status. Materials and Methods. A systematic review was conducted in PubMed, Cochrane Library, Scopus and Science Direct databases. After titles, abstracts and full-text examination, only 27 articles were selected on the basis of inclusion criteria and consequently included for quality assessments and data extraction. Results. Most of the studies reported a higher prevalence of caries experience, a poorer periodontal health and more difficulties in accessing dentalcare services among migrant groups compared with the non-migrant population. Inequalities were mostly associated with ethnic background, economic condition and social grade. Conclusion. Our review demonstrates the lack of dental health among migrants, underlining that their cultural beliefs and their social and economic living conditions could influence their oral health.

## 1. Introduction

According to the 2017 International Migration Report, the number of international migrants reached 220 million in 2010 and 258 million in 2017, showing a continuous growth in recent years [1]. Migrants represent 3.5% of the world’s population (updated to 2019) and India has the highest number of individuals living abroad [2]. Europe, Asia and Northern America host two thirds of international migrants, mainly originating from middle- and low-income countries [3,4]. In particular, a total of 4.2 million people immigrated to one of the European Union (EU) Member States during 2019 (30% of who comes from non-EU countries). In the same year, the largest total number of immigrants was reported by Germany, followed by Spain, France and Italy [5]. The reasons that prompt people to move are known: economic inequality, political instability, increased globalization [6], and it has been demonstrated that immigration status is one of the main determinant in health disparities [7,8,9]. Several factors contribute to defining migrants as vulnerable subjects: health risks before, during and after migration, different disease pro-file from that of the population of the receiving countries and barriers in accessing health care services in hosting nations [10]. Difficulties in understanding the spoken language, different cultural habits, employment problems, low socio-economic position and lack of medical insurance are conditions that may affect migrants’ general health, including their oral health status [11,12,13,14]. The risk of a poorer oral health among migrants compared to the host population has been demonstrated in the literature [15,16], even though information about this topic remains contradictory. Studies from Germany and Spain, included in the systematic review by Pabbla et al. [17], reported higher dental caries experience (DMFT Index) in migrants adolescents compared to the host population, but, on the contrary, re-searchers performed in United Kingdom (UK), Denmark and Sweden showed a lower DMFT Index among non-native subjects compared to the native population. Al-merich-Silla et al. demonstrated that immigration status and social class were significantly associated with higher caries level in immigrant children compared to Spanish children of the Valencia region [18]. DMFT score was also analyzed in migrant children attending schools in Heidelberg areas of disadvantaged socioeconomic status and reported to be significantly higher compared to non-migrants [19]. The cross-sectional study by Brzoska et al. [20] associated the scarce use of regular dental checkups by immigrants in Germany (36% lower chance than non-migrants) with their lower socio-economic status (SES), poor social support and lack of regular health insurance. Hagenfeld et al. [21] compared two migrants groups coming from Turkey and the Soviet Union with the German native population, recording a higher prevalence of maximal periodontal pocket depth above 5 mm and a lower use of dentalcare services in migrants. Therefore, migrants’ poor oral health and difficulties in accessing dental care are related to their sociodemographic and socioeconomic characteristics: low income, education level, language barriers, religious affiliation and cultural habits belonging to the country of origin [15,22]. Dental treatments in hosting countries are often perceived as more expensive compared to those in home countries [23,24]. Oral health knowledge and beliefs are generally poor among migrants, as demonstrated by Skeie et al.: South Asian and Muslim populations in Norway give no importance to oral hygiene practices and believed that deciduous teeth are not important for the oral health of their children [25].

Quality of life could be affected by poor oral health, since it may interfere with everyday activities, such as eating and talking and it may increase the risk of developing chronic diseases: periodontal microorganisms can contribute to the onset of diabetes or cardiovascular diseases and protracted oral pain can lead to nutrition problems [26,27,28,29].

For all these reasons, intervention strategies aimed at improving the oral health condition of migrants population are required [30,31].

The objectives of our systematic review were the following:

1. What are the oral health conditions among migrants from middle- and low-income countries to Europe?
2. Considering the sociodemographic (ethnic background) and socioeconomic characteristics (income, social grade, professional status) of migrants, is there an association between these variables and migrants oral health status?

Clinical Question (PICO)

- P: A sample of migrants from middle- and low-income countries to Europe
- I: Analysis of the oral health condition, oral health habits, attitude towards oral health and use of dentalcare services
- C: Association between oral health condition, oral health habits, attitude to-wards oral health and use of dentalcare services and sociodemographic/socioeconomic characteristics
- O: Presence of dental caries, periodontal status, need for dental treatment, self-reported oral health, oral health habits, oral hygiene practices, impact of the oral health on life quality

## 2. Materials and Methods

### 2.1. Protocol and Registration

Methods and inclusion criteria were selected following the PRISMA statement [32], since it provides a suitable protocol for systematic reviews.

### 2.2. Eligibility Criteria

Inclusion and Exclusion criteria

All the items concerning the oral health status in a population of migrants from middle and low-income countries to Europe were selected and included in our research. Pa-per selection was based on the following inclusion criteria:

o The selected population sample had to include subjects identified as migrants
o Studies which assessed the social fragility of the migrants’ selected subjects, by analyzing their socioeconomic characteristics (education level/professional status/money income/social class) or by identifying them as refugees or asylum seekers
o Articles which reported quantitative or qualitative data about the oral health status of the migrants included participants
o Papers written in English

Reviews and case reports were not selected and studies published before 2010 were excluded from our review, in order to collect the most recent data available in the literature.

#### 2.2.1. Electronic Search

The databases of PubMed, Cochraine Library, Science Direct and Scopus were used to conduct electronic research, selecting relevant articles (published from 2010 to date) concerning the oral health status of migrants from middle- and low-income countries to Europe. Only articles written in the English language were considered, but no restrictions were imposed with regard to the age range of the participants and to the oral health evaluation methodology. Both items with or without non-immigrant (native) population control group were included. The keywords, with the Boolean term “AND”, used for the electronic search in each database were “oral health status”, “migrants”, “oral health inequalities”, and “migration to Europe”.

#### 2.2.2. Study Selection and Data Collection Process

Eligible articles were selected following the inclusion and exclusion criteria mentioned above by two independent reviewers, who analyzed the titles, abstracts and full text of all the articles that were found during the electronic search. Disagreements between reviewers were resolved by consensus. Data collection was performed by one researcher, who extracted from each article the following information: (a) design of the study (cross-sectional, prospective/retrospective longitudinal), (b) European country in which the study was conducted (Finland, Germany, Greece, Italy, Netherlands, Norway, Spain, Sweden and UK), (c) participants’ sociodemographic characteristics (age, gender, country of origin, religious affiliation, place of residence), (d) participants’ socioeconomic status (education level, social class, marital status, monthly net income, professional status), (e) methodology used for the oral health evaluation (clinical indices/parameters, self-reported questionnaires or oral interviews); (f) quantitative/qualitative data about the oral health condition of the included subjects (dental caries, periodontal status, oral health habits, oral hygiene practices, impact of the oral health on life quality) were also extracted and used as outcome measures (means and percentages). Furthermore, the researcher collected information regarding the (g) association between the oral health parameters and the sociodemographic (ethnic background) and socioeconomic (income, social grade, professional status) characteristics of the migrant population sample, reporting them as descriptive outcomes.

## 3. Results

### 3.1. Critical Appraisal

The JBI Critical Appraisal Tool [33] was used in order to evaluate the methodological quality of the included items (Table 1, Table 2, Table 3 and Table 4) and to determine the risk of bias in their design, conduct and analysis. The JBI for case-control studies judges each study based on nine items: (1) target population, (2) participants selection methods, (3) sample size, (4) description of study subjects and setting, (5) response rate of participants, (6) diagnostic methods, (7) standardized and reliable way of measurements, (8) statistical analysis, (9) management of the participants’ response rate. Cohort studies are investigated by the same tool based on 11 items: (1) population recruitment, (2,3) exposure, (4,5) confounding factors, (6,7) outcome, (8,9,10) follow-up, (11) statistical analysis. Authors indicate for each item “yes”, “no”, “unclear”, “not applicable” and finally giving an overall appraisal.

Most of the cross-sectional studies included an appropriate sample to address the target population, sampled participants in an appropriate way, choose an adequate sample size and described subjects and settings in detail [34,35,36,37,38,39,40,41,42,44,45,46,48,49,50,51,52,53,54,55]. Only two articles [43,47] did not select an adequate sample size and one research [43] did not describe subjects in detail. None of the included items indicated the response rate, except for two articles [35,43]. Only three of the selected research papers did not provide appropriate statistical analysis [43,47,48], while all the studies used standardized and reliable methodologies for condition identification and measurement.

The exposure measurements were similar for both exposed and unexposed group and statistical analysis was appropriate in all the included cohort studies [56,57,58], but confounding factors were not identified in any of these articles.

### 3.2. Study Selection and Characteristics

**Figure 1:**
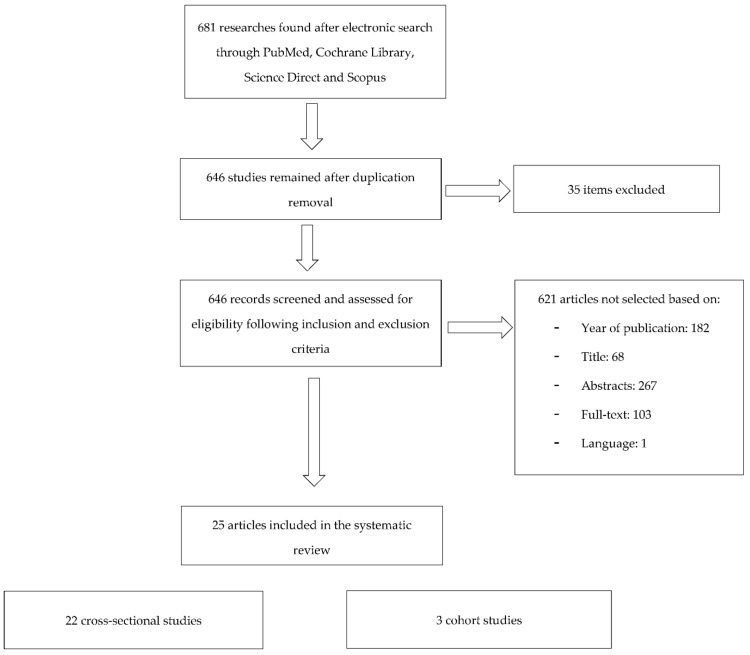
Flow chart of publication assessment.

During the electronic search on PubMed, Cochrane Library, Scopus and Science Direct databases, a total of 681 articles were found. After duplication removal, 646 items were identified and consequently subjected to titles, abstracts and full-texts examination. Only 25 items (22 cross-sectional, 1 prospective longitudinal and 2 retrospective longitudinal) were selected on the basis of inclusion criteria and included for quality assessment and data extraction: 184 studies were not selected based on the publication date (prior to 2010), 72 citations were not included after analyzing titles, 391 after reading abstracts and full-texts (absence of sociodemographic/socioeconomic status assessment, non-representative sample size, quantitative/qualitative data about oral health not re-ported) and 1 study was excluded because it was written in German language. The flow chart of publication assessment is showed in Figure 1.

The list of the included studies is presented in Table 5, Table 6 and Table 7. For each item, several information were reported: author, publication date, country in which the research was conducted, study design, number and age range of the included mi-grants (MI) subjects, investigation method used for sociodemographic (SDS) and socioeconomic status (SES) assessment, clinical and qualitative oral health parameters evaluated, statistical test used to establish the association between the oral health and the SDS/SES of the selected subjects (Table 8).

Our review included in total 138,607 participants, of which 26,277 were MI and 112,330 were non-migrants (NMI). Country of origin of MI subjects were Africa, Asia, Central and South America and Eastern Europe. The following sociodemographic characteristics of each MI participant were reported: age, gender, religious affiliation and country of origin. Socioeconomic status was also investigated on the basis of education level, social class, marital status, monthly net income, and professional status.

The oral health condition of the selected sample was analyzed using different parameters. The main oral pathologies evaluated by performing clinical oral examination were:

(1) Dental caries
  - Decayed Missing Filled Teeth Index/decayed missing filled teeth index (DMFT/dmft)
  - Decayed Missing Filled first permanent molars (DMFM)
  - Decayed Missing Filled Surfaces (DMFS)
  - Early Childhood Caries (ECC)
  - Number of teeth with untreated caries into dentine
(2) Periodontal status:
  - Approximal Plaque Index (API)
  - Debris Index Simplified (DI-S)
  - Papillary Bleeding Index (PBI)
  - Plaque Index (PI) by Silness and Loe (1964)
  - Gingival status and bleeding on gentle probing (Loe and Silness 1963)
  - Eichner’s Index
(3) Others:
  - Presence of natural teeth
  - Presence of denture
  - Unmet Treatment Needs (UTN)
  - Presence of dental trauma
  - Dean’s Index for enamel fluorosis

Questionnaires, face to face interview and phone interviews were conducted in order to investigate self-reported oral health, use of dental care services, oral hygiene habits and oral health related quality of life (OHRQoL). Due to the heterogeneity of methodologies used for the oral health condition assessment, results were reported in descriptive way.

### 3.3. Results of Individual Studies

Quantitative data about the oral health of the MI population sample are reported in Table 9 and Table 10. Results grouped by single country are presented in Table 11, Table 12, Table 13, Table 14 and Table 15. DMFT/dmft Index was the most used parameter to assess the presence of dental caries [34,35,38,41,42,43,44,46,50,52]. Periodontal health was evaluated using Approximal Plaque Index (API), Simplified Debris Index (DI-s), Papillar Bleeding Index (PBI), Plaque indices grades 2 and 3 (Silness and Loe 1964) (PLI) and Gingival indices grades 2 and 3 (gingival bleeding on gentle probing, Loe and Silness, 1963) [34,42,45,51,52].

The DMFT of MI and NMI in the research by Aarabi et al. [34] were equal to 24.8 ± 3.9 and 23.4 ± 4.6, respectively (*p* value 0.093): the number of missing teeth (M) was similar in both groups, while the number of decayed teeth (D) was on average three times higher in MI subjects. After adjusting for gender, age, monthly net income and education, the number of decayed teeth in MI was higher than NMI. The higher values of API and PBI in MI group (API = 55.3 ± 32.3, *p* value 0.002; PBI = 46.3 ± 21.1, *p* value 0.016) demonstrate that the latter had a poorer oral hygiene compared with the native control group (API = 33.0 ± 28.2, *p* value 0.002; PBI = 30.5 ± 4.5, *p* value 0.016). 

Delgado-Angulo et al. [38] associated the DMFT Index with ethnicity, nativity status and socio-economic position (SEP): Black and Asian MI had lower DMFT than White British and ethnic differences in DMFT remained significant after adjusting for SEP measures. Among MI, the higher the age of arrival and the longer the residence in the UK, the greater the DMFT (adjusted RR: 1.03 and 1.04 per additional year).

Marcenes et al. [46] highlighted that White European, Bangladeshi and Pakistani children had significantly higher dmft scores and number of untreated caries into dentine (mean dmft: 2.56, 1.25 and 1,39 respectively; mean dt: 1.91, 1.05, 1.11 respectively) than White British individuals (mean dmft: 0,60; mean dt: 0.56).

The number of decayed and filled teeth in MI children in the study by Ferrazzano et al. [41] were significantly higher (2.49 ± 1.98 and 0.56 ±1.10, *p* value < 0001) than those in NMI children (1.16 ± 1.35 and 0.38 ± 1.98, *p* value < 0001) also after adjusting for the educational level of the mothers. The unmet restorative treatment needs (UTN) in native children were lower compared to MI children (68.4% and 86.3% respectively).

Higher odds ratio of caries prevalence was found to be associated with higher age, immigrant background (OR = 2.65–4.40) and with living in lower income areas (OR = 1.34–1.72) in the article by Gatou et al. [42].

The mean DMFT of the 102 MI included by Goetz et al. [43] was equal to 6.89 ± 5.5 and only 13.7% of the refugees had a healthy dentition.

Høyvik et al. [44] registered a mean DMFT of 10.7 ± 6.8 in MI from the Middle East and of 5.7 ± 4.3 in African refugees. After adjusting for age, gender, origin and level of education, DMFT scores remained higher in Middle East subjects. 

Jacobsson et al. [45] analyzed the oral health status of 154 MI and 585 native Swedish participants aged 3, 5, 10 and 15 years in 1993 and 2003: the Plaque indices (PLI) and the Gingival indices (GI) were higher in all age groups among MI group, compared to the NMI one, except the 15-year-olds. Both in 1993 and 2009, significantly less 3 and 5 year-olds in the MI group were caries-free compared with native subjects of the same age.

Julihn et al. (2010) [57] selected a cohort of 15538 adolescents aged 13 years (14,160 NMI, 1378 MI) and followed them until they were 19 years of age. The authors showed that MI adolescents with foreign-born parents had statistically significantly more caries compared to NMI adolescents with both parents born in Sweden. The same research recorded a higher DMFSa increment in MI adolescents with 1 or more parents born abroad (53.9) compared to NMI individuals with both Swedish parents (34.7). After adjusting for sociodemographic and socioeconomic confounders (age at migration, maternal/paternal birth region, maternal/paternal education level, marital status, family income, social welfare allowance), the study found out that subjects from Eastern Europe had a higher risk of developing approximal caries lesions during the follow-up period compared to NMI participants (OR = 1.44 (1.12–1.85)).

In 2021 Julihn et al. [58] followed a sample of 3 year-old children until they were 7 years of age, demonstrating that children with both NMI parents (born in Sweden) had a lower caries experience at 3 and 7 years of age (0.1 ± 0.6 and 0.5 ± 1.3 respectively) than children with MI parents. The risk of caries experience at age 7 years was adjusted for household income level and, with regards to the lowest income, OR (CI 95%) of children with both parents born in Sweden was equal to 1.49 (1.37–1.63), OR of children with parents from high-medium-low human development countries (according to Human Development Index, HDI) resulted to be 2.89 (1.64–5.09), 1.69 (1.31–2.17) and 1.90 (1.14–3.15) respectively.

The global DMFT was calculated to be higher in Arab individuals (3.5 ± 3.6) than in Caucasian migrants (2.7 ± 3.6) by Riatto et al. [50].

Rouxel et al. [51] highlighted the association between Decayed Teeth (DT) and ethnicity/socioeconomic position (SEP): the predicted rate for MI children from India and Pakistan (3.71 (1.08–6.34) and 2.85 (1.85–3.85) respectively) was about 2–2.5 times higher than those for White British/Irish children aged 5 (1.51 (1.30–1.77)).

Solyman et al. [52] analyzed the oral health of refugees from Syria and Iraq living in Germany (aged 18–60 years), reporting a mean DMFT = 6.38 ± 5.058 and demonstrating that DMFT score was significantly associated with age and with education level ((Regression Coefficient −0.019, *p* value 0.037). This study also reported that 79% of the selected participants had bacterial plaque in all six sextants and that 60% of them presented calculus in at least three sextants.

According to Wigen et al. [55], a 5-year-old children in Norway had a higher risk of developing caries into dentine if they had one or both parents of non-western origin (OR = 4.8) and one (OR = 2.1) or both parents (OR = 3.0) with low education.

Results about the use of dentalcare services by MI and NMI were contradictory: two thirds of the MI population included by Aarabi et al. [34] showed difficulties in accessing dental care because of costs and language barriers, presenting a poorer oral hygiene than NMI group; a greater dental services utilization (in United Kingdom) was observed by Al-Haboubi et al. [36] among Asian subjects compared to White and Black individuals. The same authors underlined that access to dental services decreased in lower social classes. On the contrary, Asian and Black participants of the article by Arora et al. [37] declared that they attended dental clinics only if they suffered symptoms (unlike White British people) and their oral hygiene practices, after adjusting for age, sex, education level, household tenure and other confounders, were poorer than the NMI population. Likewise, asylum seekers in Germany selected by Freiberg et al. [56] visited dentists only because of localized and non-localized pain. According to the research by Erdsiek et al. [40], MI adults presented lower socioeconomic status and lower utilization of dental check-ups than NMI individuals. A generally poor oral health was also recorded by Høyvik et al. [44] in refugees from Middle East and Africa to Norway, half of which had oral impacts on daily performances.

Mattila et al. [47] evaluated the utilization of dental care services among MI and asylum seekers in Finland and found that the latter (100%) were significantly less satisfied with access to dental treatment and the quality of treatment than MI (18%). In total, 48% and 11% of the MI and asylum seekers groups, respectively, were aware of caries prevention methods, and none of the asylum seekers knew how to prevent gingival bleeding, while 7% of the MI did.

MI and NMI children in Spain between 3 and 14 years old were compared by Portero de la Cruz et al. [49]: 51.78% and 35.43% of MI and NMI children did not use dental services for over a year respectively. According to socioeconomic and demographic variables, lower social classes and 3–6-year-olds were less likely to use regular dental check-ups.

Dental hygiene was practiced once per day by 44.1% of the refugees studied by Goetz et al. [43] and only 4.9% of them visited dental clinics twice per year during childhood.

Agudelo-Suárez et al. [35]. and Van Meljeren-van Lunteren et al. [56] assessed the OHRQoL of MI population in Spain and Netherlands, respectively. Surinamese and Turkish children showed significant lower OHRQoL than native Dutch children, after adjusting for age, gender of children, caries experience, family income and education level of the mother. On the contrary, the MI group in the Spanish study reported a general low impact of oral health on quality of life.

Mustafa et al. [48] investigated toothbrushing-related perceptions of parents living in Norway with MI background and found that 40% of parents have knowledge about caries as a common disease among children and that 80% of them are aware of the importance of toothbrushing in primary teeth. Moreover, it was demonstrated that oral attitudes were more favorable among MI who had lived in Norway for more than 6 years.

Dujister et al. [39] studied the association existing between parental and family-related factors and childhood dental caries in Moroccan, Turkish and Dutch children. Lower social class was significantly associated with more external locus of control (LoC), poorer parental oral hygiene practices and lower dental self-efficacy and, moreover, Moroccan and Turkish parents presented a more external LoC compared to native Dutch parents.

## 4. Discussion

Our review aimed to assess the oral health status, oral health habits and use of dentalcare services among migrant population from middle- and low-income countries to Europe. Data collected in our review highlighted, in general, a higher prevalence of dental caries [34,42,45,46,51,57,58] and a poorer periodontal condition [34,42,45,51] in MI population compared with NMI groups. The impact of inequalities in terms of socioeconomic status have been largely studied in literature [74]. The research conducted in Sweden in 2006 [75] hypothesized and demonstrated that the low socioeconomic status could limit access to dentalcare services, contributing to the social inequalities in oral health. Consequently, if socioeconomic position is linked to health status, it can be stated that inequalities in socioeconomic position could be associated to ethnic inequalities in health [76]. Borrel et al. (USA) [77] examined the relationship between individual and socioeconomic characteristics and periodontal disease and highlighted that low income and low education level were associated with severe periodontitis among Whites and African Americans.

The MI population studied by Aarabi et al. [34] (coming from East Europe, Africa, Asia and South America) had a lower socioeconomic status, a worse oral health and higher treatment needs compare to NMI individuals.

Similarly, 38% of the participants (White British/Irish, Black and Asian) included in the research by Al-Haboubi et al. [36] belonged to the lowest social grade (semi- and unskilled manual workers, state pensioners, casual or lowest-grade workers, unemployed with state benefits only): the authors assessed that dental services use decreased with decreasing social grade.

Erdsiek et al. [40] found a lower access to dentalcare services in Germany among MI, 53.8% and 17.8% of whom had a middle and low socioeconomic status respectively. Authors confirmed that having a higher socioeconomic status was associated with greater use of dental prevention.

The review by Scheppers et al. [78] investigated the potential barriers and factors that could interfere with the access to health services among ethnic minorities: low education, social and socioeconomic status, ethnic background, lack of financial resources and family/social support, cultural perception about symptoms, differences in health beliefs, language skills and unawareness of service availability.

For instance, Portero de la Cruz et al. [49] attributed the disparities in the utilization of dentalcare between MI and native Spanish group to the cultural differences regarding the way families seek dental health care and to the lack of information about health benefits.

Gatou et al. [42] estimated that children’s ethnic background was the most strongly affecting risk factor for all the oral health parameters investigated in the study and reported that this relation became stronger when adjusted for independent variables, such as area-based income.

The higher caries prevalence proper of the MI group in the research by Ferrazzano et al. [41] was associated with language difficulties and inequalities in access to information and to health services.

Marcenes et al. [46] examined the inequalities in oral health between Whites, Blacks and Asians living in the most deprived boroughs in the Inner North East London: preschool children from Bangladesh and Pakistan presented a higher level of caries than White children (British, Eastern European), but, on the contrary, Indian children showed a lower level of caries than White children and Black individuals had similar dental health to Whites. Data obtained in this research confirmed the information provided by other authors, underlining that African countries experience a lower caries level than the United Kingdom [79].

Our review included thirteen articles analyzing the oral heath in children/adolescents with age ranging from 0 to 19 years old [39,42,45,46,48,49,50,51,53,54,55,57,58]. Almost all the studies [39,42,45,46,49,51,54,55] recorded a better oral condition in native children of the control groups compared to the MI groups. Only Mustafa et al. [47] assessed a good knowledge about the importance of oral hygiene among MI parents, showing that they had on average favorable attitudes, subjective norms and strong perception of behavioral control in relation to child tooth brushing.

The oral hygiene practices and behaviors of parents has a direct influence on their children’s oral health [80]. According to the socialization theory, family represents the primary socializing agent for children and, consequently, it is easy to explain why the latter adopt oral health-related habits [48]. Mothers and fathers with a foreign background are characterized by different cultures and tradition [45], migrating from their country of origin and facing several social and economic problems: this type of conditions may affect the general health of their children [78]. Julihn et al. [58] supported this theory, demonstrating that the social context of MI families from medium- and low-human development countries could be considered unfavorable for children’s oral health. Furthermore, Al-Haj Ali et al. [81] determined the risk factors associated with the presence of ECC among preschool children in eastern Saudi Arabia: mother’s occupation, carer’s smoking status and feeding practices.

The data about the lack of good oral health among refugees included in five of the selected items [43,44,50,52] are in line with other studies published in literature, which reported a high prevalence of dental caries, periodontal diseases and poor oral hygiene [82,83,84,85]. Refugees left their country of origin because of fear of persecution and/or could not return because they were exposed to persecutory events; they migrate to other countries carrying around weighty problems, facing racism, homelessness, economic and language difficulties [86]. In such condition, since refugees have to face more pressing problems than oral health, they show a tendency to under-utilize dental services [87,88]. 

This review highlighted, in agreement with the literature, that oral health is one of the greatest unmet health needs of migrants [89]. Since oral health strongly influences quality of life, training and education programs about oral health prevention should be implemented [90], considering individuals’ attitudes, capabilities, beliefs and cultural/ethnic background [91].

### Strengths and Limitations of the Study

Our study not only provides an overview of the oral health conditions of migrants in Europe, but also analyzed the association between the prevalence of oral pathologies and risk factors of the target population. After performing a critical appraisal, we recorded that most of the selected papers presented a very high quality with regards to sample selection, reliability of measurement methodologies and statistical analysis. However, the included articles used different methods to determine oral health status and as a consequence, the results were presented in a descriptive way. In fact, due to this heterogeneity, it was not possible to provide an appropriate statistical analysis. Furthermore, the selected items conducted their research in different European countries, presenting different social security systems and social conditions. For this reason, we considered this systematic review as an initial analysis that should be followed by another study investigating the oral health status of migrants in a single host country or countries with similar social conditions.

## 5. Conclusions

This systematic review reported a poorer oral condition in MI subjects from middle- and low-income countries to Europe. Oral health parameters were analyzed in association with ethnicity and socioeconomic status: it was demonstrated that foreign background, low income and social grade could be considered as risk factors for having a worse dental health.

The creation of prevention programs becomes of primary concern, aiming at strengthening oral health knowledge and practices among the MI population.

## Figures and Tables

**Table 1:** JBI for cross-sectional studies: population sample and study setting.

Studies	Was the Sample Appropriate to Address the Target Population?	Were Study Participants Sampled in an Appropriate Way?	Was the Sample Size Adequate?	Were the Study Subjects and Setting Described in Detail?
Aarabi et al. [34]	YES	YES	YES	YES
Agudelo-Suárez et al. 2019 [35]	YES	YES	YES	YES
Al-Haboubi et al. [36]	YES	YES	YES	YES
Aarora et al. 2019 [37]	YES	YES	YES	YES
Delgado-Angulo et al. 2018 [38]	YES	YES	YES	YES
Dujister et al. 2014 [39]	YES	YES	YES	YES
Erdsiek et al. 2011 [40]	YES	YES	YES	YES
Ferrazzano et al. 2019 [41]	YES	YES	YES	YES
Gatou et al. 2011 [42]	YES	YES	YES	YES
Goetz et al. 2018 [43]	YES	YES	NO	NO
Høyvik et al. 2019 [44]	YES	YES	YES	YES
Jacobsson et al. 2011 [45]	YES	YES	YES	YES
Marcenes et al. 2013 [46]	YES	YES	YES	YES
Mattila et al. 2016 [47]	YES	YES	NO	YES
Mustafa et al. 2020 [48]	YES	YES	YES	YES
Portero de la Cruz et al. 2020 [49]	YES	YES	YES	YES
Riatto et al. 2018 [50]	YES	YES	YES	YES
Rouxel et al. 2017 [51]	YES	YES	YES	YES
Solyman et al. 2018 [52]	YES	YES	YES	YES
Van der Tas et al. 2017 [53]	YES	YES	YES	YES
Van Meljeen-van Lunteren et al. 2019 [54]	YES	YES	YES	YES
Wigen et al. 2010 [55]	YES	YES	YES	YES

**Table 2:** JBI for cross-sectional studies: diagnosis, data analysis, response rate.

Studies	Was the Data Analysis Conducted with Sufficient Coverage of the Identified Sample?	Were Valid Methods Used for the Identification of the Condition?	Was the Condition Measured in a Standard, Reliable Way for all Participants?	Was There Appropriate Statistical Analysis?	Was the Response Rate Adequate, and If Not, Was the Low Response Rate Managed Appropriately?
Aarabi et al. [34]	YES	YES	YES	YES	NO
Agudelo-Suárez et al. 2019 [35]	YES	YES	YES	YES	YES
Al-Haboubi et al. [36]	YES	YES	YES	YES	NO
Aarora et al. 2019 [37]	YES	YES	YES	YES	NO
Delgado-Angulo et al. 2018 [38]	YES	YES	YES	YES	NO
Dujister et al. 2014 [39]	YES	YES	YES	YES	NO
Erdsiek et al. 2011 [40]	YES	YES	YES	YES	NO
Ferrazzano et al. 2019 [41]	YES	YES	YES	YES	NO
Gatou et al. 2011 [42]	YES	YES	YES	YES	NO
Goetz et al. 2018 [43]	YES	YES	YES	NO	YES
Høyvik et al. 2019 [44]	YES	YES	YES	YES	NO
Jacobsson et al. 2011 [45]	YES	YES	YES	YES	NO
Marcenes et al. 2013 [46]	YES	YES	YES	YES	NO
Mattila et al. 2016 [47]	YES	YES	YES	NO	NO
Mustafa et al. 2020 [48]	YES	YES	YES	NO	NO
Portero de la Cruz et al. 2020 [49]	YES	YES	YES	YES	NO
Riatto et al. 2018 [50]	YES	YES	YES	YES	NO
Rouxel et al. 2017 [51]	YES	YES	YES	YES	NO
Solyman et al. 2018 [52]	YES	YES	YES	YES	NO
Van der Tas et al. 2017 [53]	YES	YES	YES	YES	NO
Van Meljeen-van Lunteren et al. 2019 [54]	YES	YES	YES	YES	NO
Wigen et al. 2010 [55]	YES	YES	YES	YES	NO

**Table 3:** JBI for cohort studies: population, exposure, confounding factors.

Studies	Were the Two Groups Similar and Recruited from the Same Population?	Were the Exposures Measured Similarly to Assign People to Both Exposed and Unexposed Groups?	Was the Exposure Measured in a Valid and Reliable Way?	Were Confounding Factors Identified?	Were Strategies to Deal with Confounding Factors Stated?
Freiberg et al. 2020 [56]	NOT APPLICABLE	YES	YES	NO	NO
Julihn et al. 2010 [57]	YES	YES	YES	NO	NO
Julihn et al. 2021 [58]	YES	YES	YES	NO	NO

**Table 4:** JBI for cohort studies: outcome, follow-up, statistical analysis.

Studies	Were the Participants Free of the Outcome at the Start of the Study?	Were the Outcomes Measured in a Valid and Reliable Way?	Was the Follow-Up Time Reported and Sufficient to Be Long Enough for Outcomes to Occur?	Was Follow-Up Complete, and If Not, Were the Reasons to Loss to Follow-Up Described and Explored?	Were Strategies to Address Incomplete Follow-Up Utilized	Was Appropriate Statistical Analysis Used?
Freiberg et al. 2020 [56]	YES	YES	NOT APPLICABLE	NO	NO	YES
Julihn et al. 2010 [57]	YES	YES	YES	YES	NOT APPLICABLE	YES
Julihn et al. 2021 [58]	YES	YES	YES	YES	NOT APPLICABLE	YES

**Table 5:** List of included studies: design, aim, number of MI and NMI, age range, migrants assessed characteristics.

Study	Design and Aim	Number of MI	Number of NMI	Age Range	MI Assessed Characteristics
Aarabi et al. 2018 (Hamburg, Germany) [34]	Cross-sectional	61	51	≥60	Sociodemographic status *: age, gender, religious affiliation, family status, country of origin Socioeconomic status **: education, professional status, monthly net income
Agudelo-Suárez et al. 2019 (Spain) [35]	Cross-sectional analysis from a prospective cohort study	300	101	12–17 ≥18	Sociodemographic status *: age, gender, country of origin Socioeconomic status **: education, marital status, social class (manual, non-manual)
Al Haboubi et al. 2013 (London, UK) [36]	Cross-sectional	229	466	≥16	Sociodemographic status *: age, gender, country of origin Socioeconomic status **: social grade
Arora et al. 2019 (England, Wales, Northern Ireland) [37]	Cross-sectional	624	10,435	≥16	Sociodemographic status *: age, gender, country of origin Socioeconomic status **: household tenure, education level, number of household members
Delgado-Angulo et al. 2018 (East London, UK) [38]	Cross-sectional	1036	874	16–65	Sociodemographic status *: age, gender, country of origin SEP: education, professional status
Dujister et al. 2015 (Netherlands) [39]	Cross-sectional	57	35	5 and 6	Sociodemographic status *: children age, children gender, country of origin Socioeconomic status **: parents’ education level, family income, relationship status
Erdsiek et al. 2017 (Germany) [40]	Cross-sectional	3404	18,337	≥18	Sociodemographic status *: age, gender Socioeconomic status **: type of health insurance, measurement and categorization of Lampert et al. [59] → education level, occupational status, net equivalent income
Ferrazzano et al. 2019 (Naples, Italy) [41]	Cross-sectional	183	370	12–14	Sociodemographic status *: country of origin and other not specified Socioeconomic status **: family’s annual income
Freiberg et al. 2020 (Halle, Germany) [56]	Retrospective longitudinal	475 asylum seekers	/	No age range	Sociodemographic status *: age, gender, country of origin
Gatou et al. 2011 (Greece) [42]	Cross-sectional	739	4377	5–12	Sociodemographic status *: age, gender, place of residence Socioeconomic status **: area-based income
Goetz et al. 2018 (Schleswig-Holstein, Germany) [43]	Cross-sectional	102 refugees in reception centers/collective living quarters	/	16–64	Sociodemographic status *: age, gender, country of origin
Høyvik et al. 2019 (Norway) [44]	Cross-sectional	132 refugees/asylum seekers	/	>18	Sociodemographic status *: age, gender, country of origin Socioeconomic status **: education level
Jacobsson et al. 2011 (Jönköping, Sweden) [45]	Cross-sectional	154	585	3/5/10/15	Sociodemographic status *: age, gender, country of origin Socioeconomic status **: education level
Julihn et al. 2010 (Sweden) [57]	Retrospective longitudinal	1378	14,160	13 and 19 (6 years of follow-up)	Sociodemographic status *: age, gender, country of origin Socioeconomic status **: parents’ marital status, parents’ education level, social welfare allowance, family income
Julihn et al. 2021 (Sweden) [58]	Prospective longitudinal	10,180	44,491	3 and 7 (4 years of follow-up)	Sociodemographic status *: age, gender, country of origin Socioeconomic status **: parents’ marital status, parents’ education level, social welfare allowance, family income
Marcenes et al. 2013 (Tower Hamlets, Hackney and Newham, London, England) [46]	Cross-sectional	89% of 2434 included subjects	10,94% of 2434 included subjects	3–4	Sociodemographic status *: age, gender, country of origin
Mattila et al. 2016 (Finland) [47]	Cross-sectional	9 asylum seekers 29 migrants studying at the Oulu Adult Education Centre and PASK-Adult Education Centre	/	17–53	Sociodemographic status *: age, gender, country of origin Socioeconomic status **: education level
Mustafa et al. 2020 (Norway) [48]	Cross-sectional	466	/	Mothers and fathers of 0–6 months old children	Sociodemographic status *: parental age, country of origin Socioeconomic status **: parents’ education level, employment status
Portero de la Cruz et al. 2020 (Spain) [49]	Cross-sectional	253	4315	3–14	Sociodemographic status *: age, gender, country of origin Socioeconomic status **: type of household social class, size of town of residence
Riatto et al. 2018 (Melilla, Spain) [50]	Cross-sectional	156 Syrian refgees children living at the Center for Temporary Stay of MI	/	5–13	Sociodemographic status *: age, gender, religious affiliation country of origin
Rouxel et al. 2017 (England, Wales and Northern Ireland) [51]	Cross-sectional	1460	7081	5/8/12/15	Sociodemographic status *: age, gender, output area classification, country of origin Socioeconomic status **: index of Multiple Deprivation (IMD), analysis of children’s school (deprived or not deprived school, eligibility for free school meals)
Solyman et al. 2018 (Berlin, Germany) [52]	Cross-sectional	386 refugees living in reception centers/shelters/private practices	/	18–60	Sociodemographic status *: age, gender, country of origin Socioeconomic status **: education level
Van der Tas et al. 2017 (Netherlands) [53]	Cross-sectional	1618	3446	6	Sociodemographic status *: age, gender, country of origin Socioeconomic status **: parental education level, parental employment status, net household income, single parenting teenage pregnancy
Van Meljeen-van Lunteren et al. 2019 (Rotterdam, Netherlands) [54]	Cross-sectional	611	2510	9	Sociodemographic status *: age, gender, country of origin Socioeconomic status **: maternal education level, household income, generational status
Wigen et al. 2010 (Norway) [55]	Cross-sectional	70	453	5	Sociodemographic status *: parents’ age/gender, country of origin Socioeconomic status **: parents’ education level

MI = migrants; NMI = non migrants; SEP = socio-economic position. * Sociodemographic characteristics: age/gender/religious affiliation/country of origin. ** Socioeconomic characteristics: education level/social class/marital status/net income/professional status.

**Table 6:** MI and NMI country of birth, quantitative oral health indicators (QnOHI) and data collection of the included studies.

Study	MI Country of Birth	NMI Country of Birth	QnOHI	Data Collection
Aarabi et al. 2018 [34]	36: Europe 25: Africa/Asia/America	51: Germany	• DMFT according to Barmes [60]	Clinical oral examination
Agudelo-Suárez et al. 2019 [35]	126: Ecuador 122: Colombia 52: Morocco	101: Spain	/	/
Al Haboubi et al. 2013 [36]	193: Africa/Caribbean/Other 36: India/Bangladesh/Pakistan/Other	466: British/Irish/Other	/	/
Arora et al. 2017 [37]	272: India 165: Pakistan or Bangladesh 187: Black	10.435: White British	Presence of natural teeth Presence of filled teeth Presence of denture	ADHS 2009 Model [61]
Delgado-Angulo et al. 2018 [38]	1036: Africa/Caribbean/Pakistan/India/Bangladesh/Asia	874: UK	• DMFT	Clinical oral examination following UK ADHS protocol 1998 [62]
Dujister et al. 2015 [39]	31: Morocco 26: Turkey	35: Netherlands	• DMFT	Records from the pediatric dental center in the Haque (Netherlands): data were collected performing clinical oral examination
Erdsiek et al. 2017 [40]	3404: MI	18337: Germany	/	/
Ferrazzano et al. 2019 [41]	183: Eastern Europe/Asia/Africa/Turkey/South and Central America	370: Italy	• DMFT	Clinical oral examination
Freiberg et al. 2020 [56]	187: Syria 46: Afghanistan 38: Iran 29: Somalia 21: Guinea-Bissau 21: Russia 18: Eritrea 14: India 14: Kosovo 11: Benin 76: unknown/others	/	/	/
Gatou et al. 2011 [42]	739: MI	4377: Greece	dmft UTN DI-S	Clinical oral examination
Goetz et al. 2018 [43]	25: Afghanistan 19: Iraq 15: Syria 14: Eritrea 11: Yemen 7: Armenia 5: Somalia 4: Iran 2: Chechnya	/	• DMFT	Clinical oral examination
Høyvik et al. 2019 [44]	45: Middle East (Syria/Iran/Iraq/Afghanistan) 87: Africa (Eritrea/Somalia/Sudan/Nigeria)	/	• DT	Clinical oral examination by Singh et al. [63]
Jacobsson et al. 2011 [45]	154: Asia/Africa/South America/North America/Scandinavia/European countries	585: Sweden	Number of teeth dfs/DFS GI PLI	Clinical and radiographic examination
Julihn et al. 2010 [57]	140: Western Europe 315: Eastern Europe 595: Asia 143: Africa 185: South America	14160: Sweden	• DMFSa	Data were provided by Public Dental Health Service, private practicioners and the Department of Dental Medicine, Division of Pediatric Dentistry at Karolinska Institutet
Julihn et al. 2021 [58]	2363: Africa/India 7351: Eastern Europe/South America/China/Asia/Vietnam/Oceania 872: Western Europe/South Europe/North America/Korea	44491: Sweden	• Presence of caries into dentin	Clinical and radiographic examination
Marcenes et al. 2013 [46]	1.94%: White Eastern Europe 2.74%: White other 15.6%: Black Africa 7.30%: Black Other 7%: India 30.11%: Bangladesh 6.36%: Pakistan 5.14%: Asian Other 4.04%: Middle East	10.94%: White British	dmft Number of teeth with untreated caries into dentin % of children with one or more tooth with untreated caries into dentin % of children with caries experience	Clinical oral examination
Mattila et al. 2016 [47]	9 asylum, seekers: Asia 12 MI: Asia 7 MI: Africa 10 MI: Europe	/	/	/
Mustafa et al. 2020 [48]	32: Afghanistan 17: Azerbaijan/Bangladesh/Pakistan 4: Bosnia and Herzegovina 1: Dominican Republic 18: Philippines 2: Belarus 23: India 2: Indonesia 15: China 2: Kosovo 34: Lithuania 3: Moldova 2: Nepal 12: Romania 7: Russia 10: Srijlanka 1: Taiwan 10: South America 130: Africa	/	/	/
Portero de la Cruz et al. 2020 [49]	253: MI (nationality not specified)	4315: Spanish	/	/
Riatto et al. 2018 [50]	100: Arabian ethnicity 56: Caucasian ethnicity	/	• DMFT	Clinical oral examination
Rouxel et al. 2017 [51]	335: Black African and Caribbean 431: Pakistan/Bangladesh 142: India 552: Other White/Mixed White	7081: Britain/Ireland	DFT Presence of plaque Gingivitis	Children’s Dental Health Survey (CDHS) 2013
Solyman et al. 2018 [52]	239: Syria 147: Iraq	/	DMFT Dental trauma Dean’s Index (enamel fluorosis) Need of treatmentPresence of plaque Presence of calculus	Clinical oral examination
Van der Tas et al. 2017 [53]	1618: Non-Western	3446: Netherlands	• dmft	Clinical oral examination
Van Meljeen-van Lunteren et al. 2019 [54]	Mothers’ country of birth: 143: Indonesia 104: Morocco 195: Suriname 169: Turkey	Mothers’ country of birth: 2110: Netherlands	/	/
Wigen et al. 2010 [55]	Parents’ country of birth 70: Turkey/Asia/Africa/South America/Central America/Eastern Europe	Parents’ country of birth 453: Netherlands	• dmft	Clinical oral examination

ADHS 2009 = Adult Dental Health Survey 2009; API: Approximal Plaque Index; dfs = dcayed filled proximal teeth surfaces in primary dentition; DFS = Decayed Filled proximal teeth surfaces in permanent dentition; DFT = Decayed Filled permanent Teeth; DT = Decayed permanent Teeth; DI-S = Simplified Debris Index; DMFT= decayed (D), missing (M), filled (F) permanent teeth; dmft= decayed (d), missing (m), filled (f) primary teeth; DMFM = decayed, missing, filled first permanent molars; DMFSa = decayed, missing, filled surfaces approxymal; ECC = early childhood caries: GI = gingival indices; N = number; NICE = National Institute for Health and Clinical Excellence: PBI = Papillary Bleeding Index; PI = Plaque Index; PLI = Plaque indices grades 2 and 3 (Silness and Loe 1964); pufa index = pulpal involvement, ulceration, fistula and abscess in severe decayed primary teeth; UTN = Unmet Treatment Needs.

**Table 7:** Qualitative oral health indicators and data collection of the included studies.

Study	MI Country of Birth	NMI Country of Birth
Aarabi et al. 2018 [34]	Use of dental care services/barriers Oral hygiene behavior	Face to face interview: 18 questions corresponding to the German Oral Health Sruvey (DMS) IV (Micheelis and Schiffner 2006)
Agudelo-Suárez et al. 2019 [35]	OHRQoL Self-perceived dental caries/gingival bleeding/use of oral health services	OHIP-14 instrument [64]: 14 questions on impact of oral condition on people’s quality of life
Al Haboubi et al. 2013 [36]	• Use of dental care services (NICE guidelines)	Home interview with a structured questionnaire
Arora et al. 2017 [37]	Use of dental care services Self-reported oral health	ADHS 2009 model [61]
Dujister et al. 2015 [39]	Parents’ dental health efficacy Dental health-related Locus of control (Loc)	Validate questionnaire by Pine et al.
Erdsiek et al. 2017 [40]	• Use of dental check-ups in the 12c months prior to the interview (dichotomous variable)	Secondary analysis from the cross-sectional telephone survey “German Health Update 2010” by Robert Koch Institute [65]
Freiberg et al. 2020 [56]	• Dental healthcare utilization	Handwritten medical reports at Dental Department at Martin-Luther-University Halle-Wittenberg (Halle, Germany) from 1 January 2015 to 31 December 2015
Goetz et al. 2018 [43]	Year of last dental visit Regular visits to a dentist during childhood Daily dental hygiene/access to dental hygiene products Oral pain	Questionnaire
Høyvik et al. 2019 [44]	Self-perceived oral health Dental habits OIDP	Oral questions for self-perceived oral health/utilization of dental services Opened questions about dental habits Questionnaire for OIDP with 8 questions
Mattila et al. 2016 [47]	Oral health and use of dental care services Oral health related habits Dental fear	Interview of 30 min with closed and opened questions
Mustafa et al. 2020 [48]	• Parental oral health behaviors Following the Aizen’s Theory of Planned Behavior (TPB) [66]; Intention to brush child’s teeth twice a day Subjective norms towards child’s toothbrushing twice a day Perceived behavioral control Based on health belief model [67]: • Indulgence	Face to face interview of 15–20 min
Portero de la Cruz et al. 2020 [49]	Use of dental services Dental problems	Spanish National Health Survey 2017 [68]
Solyman et al. 2018 [52]	Knowledge of toothbrushing and flossing Attitude towards oral health practices of oral hygiene	Questionnaire proposed by WHO consisting of 11 opened questions [69]
Van Meljeen-van Lunteren et al. 2019 [54]	• OHRQoL	COHIP-ortho/COHIP-11
Wigen et al. 2010 [55]	Parents’ oral health behavior Parents’ attitude to oral health	Questionnaire

COHIP-11/ortho = Child Oral Health Impact Profile; OHRQoL = Oral Health Related Quality of Life; OIDP = oral impact on daily performance.

**Table 8:** Assessment of sociodemographic/socioeconomic status (SDS/SES), association between SDS/SES and quantitative/qualitative oral health indicators (QnOHI/QlOHI).

Study	Assessment of SDS	NMI Country of Birth	Association between SDS/SES and QnOHI of MI	Association between SDS/SES and QlOHI of MI
Aarabi et al. 2018 [34]	Non specified: face to face interview	Non specified: face to face interview	Logistic regression adjusted for gender, age, monthly net income, education: OR (95% CI) were reported	Logistic regression adjusted for gender, age, monthly net income, education: Coefficient (95% CI) were reported
Agudelo-Suárez et al. 2019 [35]	Structured questionnaire [70]	Based on: Social class classification → Spanish National Classification of Occupations 2011 using neo-Weberian and neo-Marxist approaches (Domingo-Salvany et al. 2013 [71])	/	Multivariate logistic regression analyses: association between SDS/SES and OHIP-4 dimension: - Unadjusted (crude OR) Unadjusted OR by age, education, marital status, social class Adjusted OR for oral health variables
Al Haboubi et al. 2013 [36]	Home interview with a structured questionnaire	Home interview with a structured questionnaire	/	Poisson regression models with robust variance: PR (95% CI) were reported
Arora et al. 2017 [37]	ADHS 2009 model [61]	ADHS 2009 model [61]	Logistic regression models adjusted for age, sex, education level, housing tenure, area socioeconomic deprivation quintile, area of residence	Logistic regression models adjusted for age, sex, education level, housing tenure, area socioeconomic deprivation quintile, area of residence
Delgado-Angulo et al. 2018 [38]	Supervised questionnaire	Supervised questionnaire: Education and the National Statistics Socio-Economic Classification (NS-SEC) were used for SEP indicators	Negative binomial regression adjusted for ethnicity, SEP, sex, age	/
Dujister et al. 2015 [39]	Self-report validate questionnaire	Self-report validate questionnaire	/	Logistic regression analysis: association of parental and family-related variables with the dental condition
Erdsiek et al. 2017 [40]	Secondary analysis from the cross-sectional telephone survey “German Health Update 2010” by Robert Koch Institute [65]	Secondary analysis from the cross-sectional telephone survey “German Health Update 2010” by Robert Koch Institute [65]	/	Multiple logistic regression models adjusted for age, gender, socioeconomic status, type of insurance
Ferrazzano et al. 2019 [41]	Questionnaire	ISEE certification for family’s annual income	One-way ANOVA test: association between DMFT and mothers’ education level	/
Freiberg et al. 2020 [56]	Handwritten medical reports at Dental Department at Martin-Luther—University Halle-Wittenberg (Halle, Germany) from 1 January 2015 to 31 December 2015	Handwritten medical reports at Dental Department at Martin-Luther—University Halle-Wittenberg (Halle, Germany) from 1 January 2015 to 31 December 2015	/	/
Gatou et al. 2011 [42]	Schools’ archives	Ministry of Economy and Finance, based on the household’s income statements of 2006	Binary logistic regression for caries prevalence adjusted for age, gender, ethnic background, residence area, area-based income: OR (95% CI) were reportedOrdinal logistic regression for DMFT/dmft adjusted for age, gender, ethnic background, residence area, area-based income: OR (95% CI) were reported	/
Goetz et al. 2018 [43]	Questionnaire	/	/	/
Høyvik et al. 2019 [44]	Not specified	Not specified	Multiple linear regression for OIDP adjusted for age, gender, education level	Multiple linear regression for DMFT/DT adjusted for age, gender, education level
Jacobsson et al. 2011 [45]	Not specified	Not specified	Logistic regression for dental caries adjusted for age, gender, parents’ education level: OR (95% CI) were reported	/
Julihn et al. 2010 [57]	Swedish National Registers	Education National Register (for parents’ education level) Total Enumeration Income Register for social-welfare allowance family income	Bivariate logistic regression analysis for DMFD adjusted for age, gender, parents’ country of birth, parents’ marital status, parents’ education level, social welfare allowance income. OR (95% CI) were reported	/
Julihn et al. 2021 [58]	Swedish National Board of Health and Welfare and by Statistics Sweden (SCB) registries	Swedish National Board of Health and Welfare and by Statistics Sweden (SCB) registries	Binary logistic regression for deft adjusted by gender, maternal age, number of children, household income level: OR (95% CI) were reported	/
Marcenes et al. 2013 [46]	School records	/	Poisson regression model for dmft/percentage of children with caries, experience adjusted by gender, borough, ethnic group: OR (95% CI) were reported	/
Mattila et al. 2016 [47]	Oral interview	Oral interview	/	/
Mustafa et al. 2020 [48]	Oral interview	Oral interview	/	/
Portero de la Cruz et al. 2020 [49]	Spanish National Health Survey 2017 [68]	Spanish National Health Survey 2017 [68]	/	Nagelkerke’s R^2^ for use of dental services adjusted by age, gender, size of town residence, type of household, social class: OR (95% CI) were reported
Riatto et al. 2018 [50]	Oral questionnaire proposed by the WHO [72]	/	Pearson correlation between oral health and children’s age	/
Roxel et al. 2017 [51]	School records	School records	Negative binomial regression model for dmft/DMFT adjusted by socioeconomic position PR (CI 95%) were reported Probit regression models for gingivitis plaque, periodontal health adjusted for socioeconomic position: PR (CI 95%) were reported	/
Solyman et al. 2018 [52]	Not specified	Not specified	Negative binomial regression model for DMFT adjusted for age, gender, education level, country for origin: Regression coefficient (standard error) was reported Ordered logistic regression for presence for plaque/presence of calculus adjusted for age, gender, education level, country of origin: OR (95% CI) were reported Multilevel mixed-effect generalized linear model for plaque/presence of calculus adjusted for age, gender, education level, country of origin: Regression coefficient (standard error) was reported	Multivariate linear regression for dental knowledge/attitude and practice adjusted for gender, age, education level, country of origin: Regression coefficient (standard error) was reported
Van der Tas et al. 2017 [53]	Questionnaire [73]	Questionnaire [73]	Multinomial logistic regression model for dmft unadjusted for parents’ education level/employment status, household income, single parenting, teenage pregnancy: OR (95% CI) were reported	/
Van Meljeen-van Lunteren et al. 2019 [54]	Questionnaire [73]	Questionnaire [73]	/	Linear regression model for OHRQoL adjusted for age, gender, family income, education level,
Wigen et al. 2010 [55]	Questionnaire	Questionnaire	Bivariate logistic regression for dmft adjusted for parents’ education level, stratified by parents’ country of birth: OR (95% CI) were reported Multiple logistic regression (Nagelkerke R2) for dmft adjusted for parents’ oral health behavior/attitude for oral health: OR (95% CI) were reported	/

CI = confidence interval.

**Table 9:** Dental caries in MI and NMI: dmft/DMFT, UTN, dsf/DFS, DMFSa. *Mean ±SD, Mean (CI 95%), %, Median (range)*.

Study	Clinical Index	MI Mean ± SD; Mean (CI 95%); %; Median (Range)	NMI Mean ± SD; Mean (CI 95%); %; Median (Range)	*p* Value
Aarabi et al. 2018 [34]	DMFT	24.8 ± 3.9	23.4 ± 4.6	0.093
Ferrazzano et al. 2019 [41]	DMFT UTN	3.92 ± 2.92 86.3%	3.29 ± 3.21 68.4%	0.027
Gatou et al. 2011 [42]	dmft/DMFT	3.68 ± 0.13/1.14 ± 0.06	1.61 ± 0.04/0.61 ± 0.02	0.001
Goetz et al. 2018 [43]	DMFT	6.89 ± 5.5	/	/
Høyvik et al. 2019 [44]	DMFT	Middle East:10.7 ± 6.8 Africa: 5.7 ± 4.3	/	0.001
Jacobsson et al. 2011 [45]	Dfs/DFS	dfs/DFS in the different age group: 3 yo = 4.5 (1.8–7.1) 5 yo = 8.5 (4.7–12.3) 10 yo = 7.0 (4.8–9.2) 15 yo = 18.1 (13.2–23.0)	dfs/DFS in the different age group: 3 yo = 0.6 (0.3–1.0) 5 yo = 2.7 (1.4–3.9) 10 yo = 5.5 (4.8–6.2) 15 yo = 18.2 (15.1–21.2)	0.008 0.006 0.196 0.985
Julihn et al. 2010 [57]	DMFSa	DMFSa in the different age group (foreign-born adolescents with ≥1 foreign-born parents): 13 yo = 0.58 ± 1.34 19 yo = 2.77 ± 4.16	DMFSa in the different age group (adolescents with two Swedish-born parents): 13 yo = 0.24 ± 0.77 19 yo = 1.31 ± 2.68	/
	DMFSa increment > 0	DMFSa increment in foreign-born adolescents with ≥1 foreign-born parents: 53.9	DMFSa in adolescents with two Swedish-born parents: 34.7	
Julihn et al. 2021 [58]	Presence of caries into dentin	Children with: one or both parents foreign-born: 6.3% from high HDI: 7.2% from medium HDI: 16.7% from low HDI: 16.8%	Children with both parents born in Sweden: 3.0%	/
Marcenes et al. 2013 [46]	dmft	Eastern European: 2.56 (1.12–3.99) Black African: 0.56 (0.26–0.87) Asian Indian: 0.84 (0.95, 1.56) Bangladeshi: 1.25 (0.94–1.83) Pakistani: 1.39 (0.24–1.07) Asian Other: 0.66 (0.04–1.10) Middle Eastern: 1.30 (0.34–2.24)	White British: 0.60 (0.29–0.92) (prevalence rate ratios (95% CI = 1))	0.001 0.85 0.30 0.01 0.004 0.85 0.09
	Number of teeth with untreated caries into dentine (dt)	Eastern European: 1.91 (0.75–3.09) Black African: 0.54 (0.23, 0.84) Asian Indian: 0.82 (0.53–1.12) Bangladeshi: 1.05 (0.80–1.29) Pakistani: 1.11 (0.83–1.40) Asian Other: 0.59 (0.20–0.99) Middle Eastern: 1.19 (0.22–2.17)	White British: 0.56 (0.25–0.87) (prevalence rate ratios (95% CI = 1))	0.006 0.89 0.28 0.04 0.03 0.91 0.12
Riatto et al. 2018 [50]	DMFT	Caucasian: 2.7 ± 3.6 Arabian: 3.5 ± 3.6	/	<0.05
Rouxel et al. 2018 [51]	DT (Decayed Teeth)	Indian: 2.83 ± 2.52	White British & Irish: 1.48 ± 2.46	/
		Pakistani: 3.04 ± 3.51		
		Bangladeshi: 2.52 ± 2.77		
		Black African: 0.81 ± 1.20		
		Black Caribbean:1.65 ± 1.52		
	FT (Filled Teeth)	Indian: 0.17 ± 0.39	White British & Irish: 0.09 ± 0.45	
		Pakistani: 0.18 ± 0.55		
		Bangladeshi 0.20 ± 0.79		
		Black African:0.31 ± 0.96		
		Black Caribbean: 0.04 ± 0.21		
Solyman et al. 2018 [52]	DMFT	6.38 ± 5.058	/	/

DFS = Decayed Filled Tooth Surfaces for Permanent Dentition; deft = decayed extracted filled primary teeth; dfs/DFS proximal = decayed filled tooth proximal surfaces; DMFSa = Decayed Missing Filled Surfaces approximal; DMFT = Decayed Missing Filled Permanent Teeth; dmft = decayed missing filled primary teeth; HDI = Human Development Index pufa index = pulpal involvement, ulceration, fistula and abscess in severly decayed primary teeth;; UTN = unment restorative treatment.

**Table 10:** Periodontal parameters in MI and NMI: API, PBI, DI-s. PLI, GI, presence of plaque and calculus on six sextants.

Study	Clinical Index	IM (Mean ± SD); Mean (CI 95%)	NIM (Mean ± SD); Mean (CI 95%)	*p* Value
Aarabi et al. 2018 [34]	API PBI	55.3 ± 32.3 46.3 ± 21.1	33.0 ± 28.2) 30.5 ± 4.5	0.002 0.016
Gatou et al. 2011 [42]	DI-s	0.94 ± 0.03	0.72 ± 0.01	0.001
Jacobsson et al. 2011 [45]	PLI	PI in the different age group: 3 yo = 13.5 (3.4–23.5) 5 yo = 13.6 (4.6–22.5) 10 yo = 53.1 (35.4–70.8) 15 yo = 31.8 (18.1–45.5)	PI in the different age group: 3 yo = 7.3 (4.2–10.3) 5 yo = 9.4 (6.7–12.0) 10 yo = 28.5 (22.3–34.7) 15 yo = 32.5 (25.8–39.2)	0.125 0.355 0.012 0.927
	GI	BoP in the different age group: 3 yo = 14.6 (7.9–21.2) 5 yo = 11.9 6.9–16.8 10 yo = 26.1 (20.2–32.0) 15 yo = 22.5 (14.7–30.4)	BoP in the different age group: 3 yo = 4.4 (3.5–5.3) 5 yo = 8.7 (6.9–19.5) 10 yo = 17.2 (14.5–20.0) 15 yo = 20.8 (16.9–24.7)	0.005 0.152 0.005 0.675
Rouxel et al. 2018 [51]	Gingivitis	Indian: 26.3% Pakistani: 25.1% Bangladeshi: 42.2% Black African: 11.9% Black Caribbean: 15.4%	White British & Irish: 23.3%	
	Plaque	Indian: 31.8% Pakistani: 50.8% Bangladeshi: 56.8% Black African: 25.4% Black Caribbean: 27.0%	White British & Irish: 32%	
Solyman et al. 2018 [52]	Presence of Plaque on six sextants	78.85%	/	/
	Presence of calculus on six sextants	29.86%	/	

API = Approximal Plaque Index; DI-S = Simplified Debris Index; GI = Gingival indices; MPS = Mucosal Plaque Index; PBI = Papillar Bleeding Index; PLI = Plaque indices grades 2 and 3 (Silness and Loe 1964).

**Table 11:** Dental caries in MI and NMI living in Germany.

Study	Clinical Index	MI Mean ± SD; Mean (CI 95%); %; Median (Range)	NMI Mean ± SD; Mean (CI 95%); %; Median (Range)	*p* Value
Aarabi et al. 2018 [34]	DMFT	24.8 ± 3.9	23.4 ± 4.6	0.093
Goetz et al. 2018 [43]	DMFT	6.89 ± 5.5	/	/
Solyman et al. 2018 [52]	DMFT	6.38 ± 5.058	/	/

DMFT = Decayed Missing Filled Permanent Teeth.

**Table 12:** Periodontal status in MI and NMI living in Germany.

Study	Clinical Index	IM (Mean ± SD); Mean (CI 95%)	NIM (Mean ± SD); Mean (CI 95%)	*p* Value
Aarabi et al. 2018 [34]	API	55.3 ± 32.3	33.0 ± 28.2)	0.002
	PBI	46.3 ± 21.1	30.5 ± 4.5	0.016
Solyman et al. 2018 [52]	Presence of Plaque on six sextants	78.85%	/	/
	Presence of calculus on six sextants	29.86%	/	

API = Approximal Plaque Index; PBI = Papillar Bleeding Index.

**Table 13:** Dental caries in MI and NMI living in United Kingdom.

Study	Clinical Index	MI Mean ± SD; Mean (CI 95%); %; Median (Range)	NMI Mean ± SD; Mean (CI 95%); %; Median (Range)	*p* Value
Marcenes et al. 2013 [46]	dmft	Eastern European: 2.56 (1.12–3.99) Black African: 0.56 (0.26–0.87) Asian Indian: 0.84 (0.95, 1.56) Bangladeshi: 1.25 (0.94–1.83) Pakistani: 1.39 (0.24–1.07) Asian Other: 0.66 (0.04–1.10) Middle Eastern: 1.30 (0.34–2.24)	White British: 0.60 (0.29–0.92) (prevalence rate ratios (95% CI = 1))	0.001 0.85 0.30 0.01 0.004 0.85 0.09
	Number of teeth with untreated caries into dentine (dt)	Eastern European: 1.91 (0.75–3.09) Black African: 0.54 (0.23, 0.84) Asian Indian: 0.82 (0.53–1.12) Bangladeshi: 1.05 (0.80–1.29) Pakistani: 1.11 (0.83–1.40) Asian Other: 0.59 (0.20–0.99) Middle Eastern: 1.19 (0.22–2.17)	White British: 0.56 (0.25–0.87) (prevalence rate ratios (95% CI = 1))	0.006 0.89 0.28 0.04 0.03 0.91 0.12
Rouxel et al. 2018 [51]	DT (Decayed Teeth)	Indian: 2.83 ± 2.52 Pakistani: 3.04 ± 3.51 Bangladeshi: 2.52 ±2.77 Black African: 0.81 ± 1.20 Black Caribbean:1.65 ± 1.52	White British & Irish: 1.48 ± 2.46	/
	FT (Filled Teeth)	Indian: 0.17 ± 0.39 Pakistani: 0.18 ± 0.55 Bangladeshi 0.20 ± 0.79 Black African:0.31 ± 0.96 Black Caribbean: 0.04 ± 0.21	White British & Irish: 0.09 ± 0.45	

DMFT = Decayed Missing Filled Permanent Teeth; dmft = decayed missing filled primary teeth.

**Table 14:** Dental caries and periodontal status in MI and NMI living Spain, Italy and Greece.

Study	Clinical Index	MI Mean ± SD; Mean (CI 95%); %; Median (Range)	NMI Mean ± SD; Mean (CI 95%); %; Median (Range)	*p* Value
Ferrazzano et al. 2019 (Italy) [41]	DMFT	3.92 ± 2.92	3.29 ± 3.21	0.027
	UTN	86.3%	68.4%	
Riatto et al. 2018 (Spain) [50]	DMFT	Caucasian: 2.7 ± 3.6 Arabian: 3.5 ± 3.6	/	<0.05
Gatou et al. 2011 (Greece) [42]	dmft/DMFT	3.68 ± 0.13/1.14 ± 0.06	1.61 ± 0.04/0.61 ± 0.02	0.001
	DI-s	0.94 ± 0.03	0.72 ± 0.01	0.001

DI-S = Simplified Debris Index; DMFT = Decayed Missing Filled Permanent Teeth; dmft = decayed missing filled primary teeth; UTN = unment restorative treatment.

**Table 15:** Dental caries and periodontal status in MI and NMI living Norway and Sweden.

Study	Clinical Index	MI Mean ± SD; Mean (CI 95%); %; Median (Range)	NMI Mean ± SD; Mean (CI 95%); %; Median (Range)	*p* Value
Høyvik et al. 2019 (Norway) [44]	DMFT	Middle East:10.7 ± 6.8 Africa: 5.7 ± 4.3	/	0.001
Jacobsson et al. 2011 (Sweden) [45]	Dfs/DFS	dfs/DFS in the different age group: 3 yo = 4.5 (1.8–7.1) 5 yo = 8.5 (4.7–12.3) 10 yo = 7.0 (4.8–9.2) 15 yo = 18.1 (13.2–23.0)	dfs/DFS in the different age group: 3 yo = 0.6 (0.3–1.0) 5 yo = 2.7 (1.4–3.9) 10 yo = 5.5 (4.8–6.2) 15 yo = 18.2 (15.1–21.2)	0.008 0.006 0.196 0.985
	PLI	PLI in the different age group: 3 yo = 13.5 (3.4–23.5) 5 yo = 13.6 (4.6–22.5) 10 yo = 53.1 (35.4–70.8) 15 yo = 31.8 (18.1–45.5)	PLI in the different age group: 3 yo = 7.3 (4.2–10.3) 5 yo = 9.4 (6.7–12.0) 10 yo = 28.5 (22.3–34.7) 15 yo = 32.5 (25.8–39.2)	0.125 0.355 0.012 0.927
	GI	BoP in the different age group: 3 yo = 14.6 (7.9–21.2) 5 yo = 11.9 6.9–16.8 10 yo = 26.1 (20.2–32.0) 15 yo = 22.5 (14.7–30.4)	BoP in the different age group: 3 yo = 4.4 (3.5–5.3) 5 yo = 8.7 (6.9–19.5) 10 yo = 17.2 (14.5–20.0) 15 yo = 20.8 (16.9–24.7)	0.005 0.152 0.005 0.675
Julihn et al. 2010 (Sweden) [57]	DMFSa	DMFSa in the different age group (foreign-born adolescents with ≥1 foreign-born parents): 13 yo = 0.58 ± 1.34 19 yo = 2.77 ± 4.16	DMFSa in the different age group (adolescents with two Swedish-born parents): 13 yo = 0.24 ± 0.77 19 yo = 1.31 ± 2.68	/
	DMFSa increment > 0	DMFSa increment in foreign-born adolescents with ≥1 foreign-born parents: 53.9	DMFSa in adolescents with two Swedish-born parents: 34.7	
Julihn et al. 2021 (Sweden) [58]	Presence of caries into dentin	Children with: one or both parents foreign-born: 6.3% from high HDI: 7.2% from medium HDI: 16.7% from low HDI: 16.8%	Children with both parents born in Sweden: 3.0%	/

DFS = Decayed Filled Tooth Surfaces for Permanent Dentition; dfs/DFS proximal = decayed filled tooth proximal surfaces; DMFSa = Decayed Missing Filled Surfaces approximal; DMFT = Decayed Missing Filled Permanent Teeth; GI = Gingival indices; PLI = Plaque indices grades 2 and 3 (Silness and Loe 1964).

## Data Availability

Not applicable.
